# Piston Error Measurement for Segmented Telescopes Based on a Hybrid Artificial Neural Network

**DOI:** 10.3390/s23208399

**Published:** 2023-10-12

**Authors:** Dan Yue, Pengcheng Song, Chongshuai Wang, Yahui Chuai

**Affiliations:** College of Physics, Changchun University of Science and Technology, Changchun 130022, China; 2021100186@mails.cust.edu.cn (P.S.);

**Keywords:** piston error, segmented telescope, artificial neural network

## Abstract

To address the difficulty and complexity of detecting piston errors for segmented telescopes, this paper proposes a new piston error measurement method based on a hybrid artificial neural network. First, we use the Resnet network to learn the mapping relationship between the focal plane degradation image and signs of the piston error. Then, based on the established theoretical relationship between the modulation transfer function and the piston error, a BP neural network is used to learn the mapping relationship between the *MTF* and the absolute value of the piston error. After the training of the hybrid network is completed, a wide-range and high-precision detection of the piston error of the sub-mirrors can be achieved using the combined output of the two networks, where only a focal plane image of the point source with broadband illumination is used as the input. The detection range can reach the entire coherent length of the input broadband light, and the detection accuracy can reach 10 nm. The method proposed in this paper has the advantages of high detection accuracy, a wide detection range, low hardware cost, a small network scale, and low training difficulty.

## 1. Introduction

In order to meet the growing demand for space exploration and to obtain a higher observation resolution, in recent years, telescopes have had larger apertures and longer focal lengths [1]. However, due to the limitations of mirror material preparation, processing, and testing, and the supporting structure, transport, and launch costs, it is difficult to build a single primary mirror with a diameter of more than 10 m at present. Segmented mirrors have been proposed to solve this problem, utilizing the segmented sub-mirrors with smaller diameters to form the larger primary mirror within the system [2]. However, for a segmented telescope, there are relative position errors among each sub-mirror, and especially for a space-segmented telescope, the relative position errors will be further aggravated under the influence of external interference, thermal deformation, gravity deformation, spacecraft jitter, and other factors, which can seriously affect the final imaging quality of the telescope [3].

The relative position errors between the sub-mirrors mainly include piston errors along the optical axis perpendicular to the sub-mirror plane, and tip–tilt errors around the two axes in the sub-mirror plane, which can be described by the first three terms of the Zernike polynomial. In order to make the imaging quality of a segmented telescope similar to that of a single primary mirror with the same aperture, the RMSE value of the relative position error between each sub-mirror is generally required to be less than 1/40 in terms of the observation wavelength. One of the most difficult problems is to correct the piston error between the sub-mirrors, since the piston error has the 2π entanglement problem of single-wavelength optical detection. 

Recently, many piston error detection algorithms have been proposed and applied to segmented telescopes. For example, the improved wideband/narrowband Shack–Hartmann method proposed by Chanan has been successfully applied to the Keck series of telescopes; the piston error detection range of the broadband Shack–Hartmann algorithm [4] is ±10λ, and the accuracy is λ/3 RMS. The piston error detection range of the narrowband Shack–Hartmann algorithm [5] is ±λ/4 and the accuracy is λ/140 RMS. Esposito et al. used a quadrangular mirror to detect the piston error [6,7], where the detection accuracy could reach 10 nm. A Mach–Zehnder interference method [8] was proposed by Angle, where a multi-wavelength light is used for detection; the detection range can be extended to several microns and the detection accuracy can reach 30 nm. When developing next-generation space telescope technology, the United States proposed a white light interferon-based dispersive fringe sensor [9,10], which is composed of a broadband light source, a splitter prism, and a grating, with a piston error detection range of ±200 um and a detection accuracy of 20 nm. However, these abovementioned wavefront detection techniques need to introduce new optical hardware equipment to the original optical system, which heavily increases the complexity of the optical path. The wavefront detection technology based on the focal plane image mainly includes phase retrieval [11,12] and the phase diversity algorithm [13,14], and has low hardware costs, since no other hardware equipment is needed. These methods have high precision but narrow detection ranges, which are not suitable for detecting large piston errors. In 2016, Junlun Jiang et al. [15,16] found that, for the point source observation target with broadband illumination, the modulation transfer function (*MTF*) of the system optical transfer function (*OTF*) has a clear mathematical relationship with the piston error of the sub-mirror, and the piecework polynomial is adapted to fit the theoretical relationship. The detection range can reach a 1/2 coherent wavelength of input broadband light and the detection accuracy is 0.026λ RMS (λ = 633 nm). However, the theoretical relationship between the *MTF* and the piston error of the system is an even function, which means that the *MTF* of a system is the same when the piston error has the same absolute value while the sign of the piston error is opposite. Therefore, this method only measures the absolute value of the piston error between the sub-mirrors and cannot identify the specific spatial relative positions of each sub-mirror.

Aimed at this problem, based on the established theoretical relation between the *MTF* and the piston error, this paper does not use the polynomial fitting method as it adopts a hybrid artificial neural network to measure the piston error. Although the system *MTF* is the same when the absolute value of the piston error is the same, despite the sign of the piston error being different, the system point spread function (*PSF*) has a significant difference. Therefore, we first use the Resnet network to learn the mapping relationship between the system *PSF* and the sign of the piston error, and then use the BP (back-propagation) neural network to learn the mapping relationship between the system *MTF* and the absolute value of the piston error; thus the precise measurement of the piston error can be realized by the combination output of the two networks. The reason for using a hybrid artificial neural network is that the system *MTF* cannot distinguish the sign of the piston error, and it is quite difficult to realize piston error detection with a wide range and with high precision directly using a single neural network from the focal plane image of the system. For example, when the detection range is [−200~200] µm and the detection accuracy is required to be 0.01 µm, for a segmented telescope with *N* sub-mirrors, the output classification of a single neural network is about 40,001*^N−1^*. When the Resnet network is used to detect the sign of the piston error, the outputs are only divided into 2*^N−1^* classes, so the network size and the training difficulty can be greatly reduced. When calculating the specific value of the piston error, since the theoretical relationship between the *MTF* and the piston value is established, a BP neural network is used to realize the high-precision detection of the absolute value of the piston error.

In the following, we describe the imaging system model, the generation of training data, and the implementation of the Resnet and BP networks used in our study in Section 2. Then, the results are presented in Section 3, including the simulation results for the 2-pupil segmented system and the 4-pupil segmented system, as well as a comparison of the results of our hybrid network and those of other single networks. Finally, concluding thoughts are offered in Section 4.

## 2. Piston Error Detection Method Based on Hybrid Neural Network

**Figure 1:**
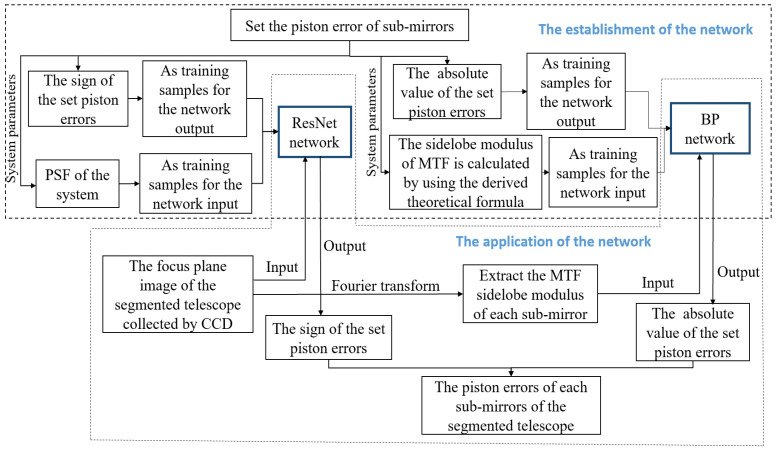
The principle of piston error detection method based on the hybrid neural network.

The principle of the piston error detection method based on the hybrid neural network proposed in this paper is shown in Figure 1. It mainly includes using the Resnet network to detect the signs of piston error from the focal plane degradation image and using the BP neural network to detect the absolute value of the piston error from the system *MTF*. In this part, we first describe the acquisition of focal plane degradation images and the establishment of theoretical relation between the system *MTF* and piston error value of sub-mirrors, then the principle and procedure of piston error detection using the hybrid neural networks are introduced. 

### 2.1. The Acquisition of Focal Plane Degradation Image

For a segmented telescope like Keck, all of the sub-mirrors are assumed to have the same shape and be perfect without high-order aberrations, except pistons and tip–tilts. Thus, the generalized pupil function (GPF) can be shown as:

(1) $$P(\varepsilon ,\eta )=\sum_{n=1}^{N}p_{n}(\varepsilon ,\eta )\exp[i\frac{2\pi }{\lambda }(e_{n}Z_{1}+t_{xn}Z_{2}+t_{yn}Z_{3})],$$

where *Z*_1_, *Z*_2_, and *Z*_3_ are the first three terms of the Zernike polynomials, *e_n_*, *t_xn_*, and *t_yn_* are the corresponding Zernike polynomial coefficients of the *n*th sub-mirror, respectively, (ε,η) is the coordinate vector in pupil plane, $p_{n}$ is the is the binary function of sub-aperture, and *N* is the total number of sub-mirrors.

Based on the principle of Fourier optics, the relationship between the point spread function (*PSF*) of the system and the GPF is:

(2) $$PSF\left(x,y,\lambda \right)={\left|ℑ\left\{P(\varepsilon ,\eta )\right\}\right|}^{2},$$

where $\left(x,y\right)$ is the coordinate vector in the image plane, λ is the wavelength of input light, and $ℑ\left\{\cdot \right\}$ denotes the Fourier transform. When the input light is not monochromatic light, and is centered at $\lambda_{0}$ with the bandwidth Δλ, the *PSF* is defined as:

(3) $$PSF\left(x,y,\lambda_{0},\Delta \lambda \right)=\int_{\lambda_{0}-\frac{\Delta \lambda }{2}}^{\lambda_{0}+\frac{\Delta \lambda }{2}}PSF\left(x,y,\lambda \right)S\left(\lambda \right)d\lambda ,$$

where $S\left(\lambda \right)$ is *PSF* weight of different wavelengths, assuming $S\left(\lambda \right)=1$.

According to the Fourier optics principle, the focal plane degradation image of the system is the convolution of the observation object and the system *PSF*, hence for an ideal point source observation target, the system focal plane image can be equivalent to the system multi-wavelength *PSF*. Combined with the three formulas given above, the focal plane degradation image of the segmented telescope for a point source observation target with broadband illumination can be acquired. The following figures show the corresponding simulation results for a segmented telescope composed of two sub-pupils.

**Figure 2:**
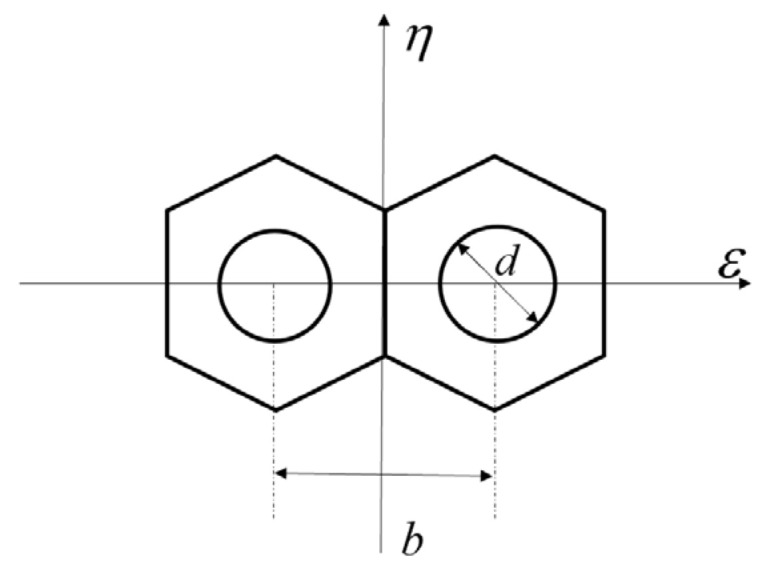
Two sub-mirrors segmented telescope with sparse circles configuration.

Figure 2 shows the segmented optics system model. A mask with two circles was set on the exit-pupil plane of the primary mirror to fragment the pupil, so the sidelobes of the system *MTF* could be separated from its main peak. Furthermore, *b* is the distance between the center of the two circle pupils on the mask and *d* is the diameter of the circle pupil. We set up this optical system in MATLAB; the sampling grid of the exit pupil plane was set as 256 × 256 pixels, the pixel size of the CCD was 3.5 μm, and the F# of the optical system was 8. Thus, the circumscribed circle diameter of the single hexagonal sub-mirror was 59 pixels, the diameter of the circle on the mask was 18 pixels, and the distance between the centers of the two circles was 52 pixels to satisfy the Nyquist sampling criterion. The central wavelength of the input broad light was 632.8 nm and its bandwidth was 20 nm. Half of the coherent length of this input broadband light is

(4) $$L=\frac{L_{c}}{2}=\frac{\lambda_{0}{}^{2}}{2\Delta \lambda }=\frac{{\left(632.8\mathrm{nm}\right)}^{2}}{2\times 20\mathrm{nm}}\approx 10\mu m.$$

The coherence length of the input broadband light determines the effective detection range of the piston error. The narrower the spectral width of the input broadband light, the longer the coherence length and the larger the piston error effective detection range of the algorithm. Thus, the effective detection range of the piston error here is [−10~10] µm.

**Figure 3:**
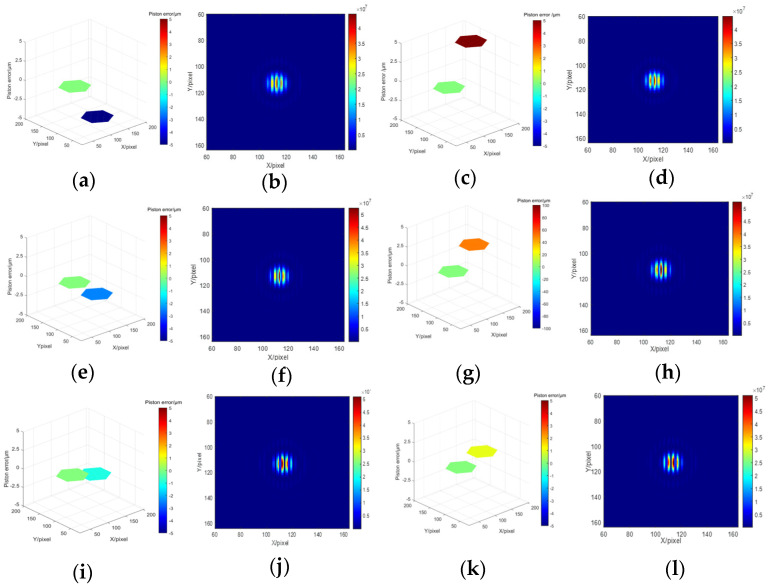
Piston errors and their corresponding system focal plane images (**a**) p = − 5 μm; (**b**) corresponding focal plane image with p = −5 μm; (**c**) p = 5 μm; (**d**) corresponding focal plane image with p = 5 μm; (**e**) p = −2.5 μm; (**f**) corresponding focal plane image with p = −2.5 μm; (**g**) p = 2.5 μm; (**h**) corresponding focal plane image with p = 2.5 μm; (**i**) p = −1 μm; (**j**) corresponding focal plane image with p = −1 μm; (**k**) p = 1 μm; (**l**) corresponding focal plane image with p = 1 μm.

Different piston errors are introduced to the right sub-pupil while the left sub-mirror is set as the reference mirror. Several groups of the introduced piston error and the corresponding system focal plane degradation images are shown in Figure 3. We can see that piston errors with the same absolute value but opposite sign corresponds to different focal plane degradation images. 

### 2.2. Theoretical Relation between System MTF and Piston Error

For a segmented telescope with a mask set on the exit-pupil plane and the observation target as a point source with broadband illumination, there is a determined theoretical relationship between the system *MTF* and the piston errors of the sub-mirrors based on Fourier optics. Junlun Jiang et al. have deduced the formula in detail and presented their work in paper [15]. Based on their work, we further deduced the theoretical relationship and briefly introduce the derivation process.

For the segmented system shown in Figure 2, the GPF is

(5) $$G(\varepsilon ,\eta )=A(\varepsilon ,\eta )\left[circ\left(\frac{\varepsilon -b/2,\eta }{d/2}\right)\cdot e^{i\phi_{1}}+circ\left(\frac{\varepsilon +b/2,\eta }{d/2}\right)\cdot e^{i\phi_{2}}\right],$$

where A(ε,η) is the binary shape function of the hexagon segment, $circ\left(\right)$ stands for circle function, the phase difference between the two segments is $\Delta \phi =\phi_{1}-\phi_{2}=\frac{2\pi }{\lambda }2p$, λ is the observation wavelength, and *p* is the piston error between the two sub-mirrors. When the input light centered at $\lambda_{0}$ has a broad spectrum Δλ, based on Equations (3) and (4), the system *PSF* can be written as

(6) $$PSF(x,y,\lambda_{0},\Delta \lambda )=\frac{\Delta \lambda }{n}\sum_{t=1}^{n}2{\left(\frac{d}{2}\right)}^{2}\frac{J_{1}{}^{2}(\pi d\sqrt{x^{2}+y^{2}})}{x^{2}+y^{2}}\left[1+\cos\left(\frac{2\pi }{\lambda_{t}}2p-2\pi xb\right)\right],$$

where $J_{1}\left\{\right\}$ is first order Bessel function and Δλ is equally divided into *n* intervals since a differential summation approximation is used to replace the integral calculation. The complex *OTF* of input broadband light is the 2D Fourier transform of the *PSF* in Equation (6), which is shown as

(7) $$\begin{array}{ll} OTF(f_{x},f_{y},\lambda_{0},\Delta \lambda ) & =ℑ\left\{PSF(x,y,\lambda_{0},\Delta \lambda )\right\}\\ & =\frac{\Delta \lambda }{n}\sum_{t=1}^{n}\left[\begin{array}{l} 2OTF_{sub}(f_{x},f_{y})+OTF_{sub}(f_{x}+\frac{b}{\lambda_{t}f},f_{y})e^{-i\frac{2\pi }{\lambda_{t}}2p}+\ldots \\ OTF_{sub}(f_{x}-\frac{b}{\lambda_{t}f},f_{y})e^{i\frac{2\pi }{\lambda_{t}}2p} \end{array}\right], \end{array}$$

where $\left(f_{x},f_{y}\right)$ is the spatial frequency in the *x* and *y* direction, respectively, and $OTF_{sub}(f_{x},f_{y})$ is the *OTF* of a single circle aperture diffraction system given by

(8) $$OTF_{sub}(f_{x},f_{y})=\left\{\begin{array}{l} \frac{2}{\pi }\left[\arccos\left(\frac{\rho }{2\rho_{0}}\right)-\frac{\rho }{2\rho_{0}}\sqrt{1-{\left(\frac{\rho }{2\rho_{0}}\right)}^{2}}\right],\;\;\;\;\;\;\;\;\;\;\;\;\;\;\;\;\rho \leq 2\rho_{0}\;\;\;\;\;\;\;\;\;\;\\ 0\;\;\;\;\;\;\;\;\;\;\;\;\;\;\;\;\;\;\;\;\;\;\;\;\;,others \end{array}\right.$$

among which $\rho =\sqrt{f_{x}^{2}+f_{y}^{2}}$ is radial coordinate on the frequency plane, $\rho_{0}=\frac{d}{2\lambda f}$ is the system cut-off frequency, and *f* is focal length of the imaging lens. Performing modulus operation to *OTF*, the *MTF* of the system can be obtained. Based on Equation (7), the sidelobes of *MTF* can be extracted out and shown as

(9) $$MTF_{sidelobe}(f_{x},f_{y},\lambda )=\frac{\Delta \lambda }{n}\left|\sum_{t=1}^{n}\left[OTF_{sub}(f_{x}+\frac{b}{\lambda_{t}f},f_{y})e^{-i\frac{2\pi }{\lambda_{t}}2p}\right]\right|.$$

Since the value of the *MTF* central peak is 1, the peak height value of the *MTF* sidelobe with piston error is

(10) $$MTF_{sidelobe}=\frac{1}{nN}\left|\sum_{t=1}^{n}e^{-i\frac{2\pi }{\lambda_{t}}2p}\right|.$$

We can see that the peak height of the *MTF* sidelobe is only related to the number of sub-pupils of the segmented telescope, the input wavelength, and the piston error between segments. So, the modulus of the *MTF* sidelobe can be easily calculated when the piston error is known. 

**Figure 4:**
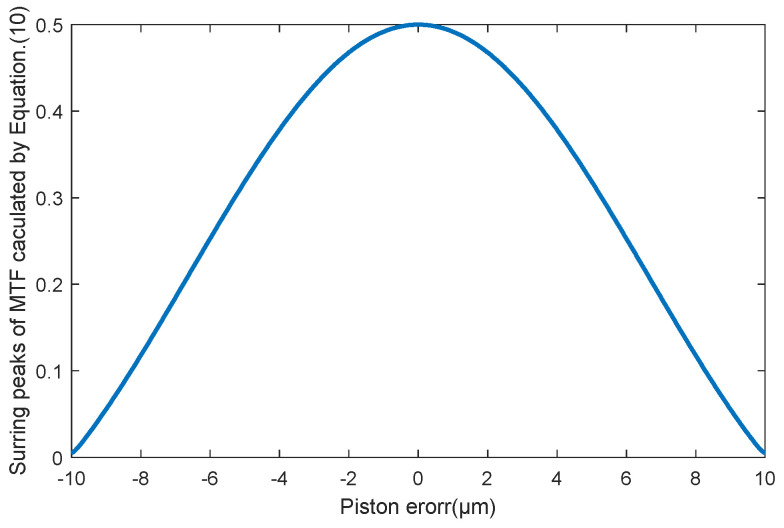
The *MTF* sidelobes calculated from Equation (10) corresponding to different piston errors of the two sub-mirrors segmented telescope system.

**Figure 5:**
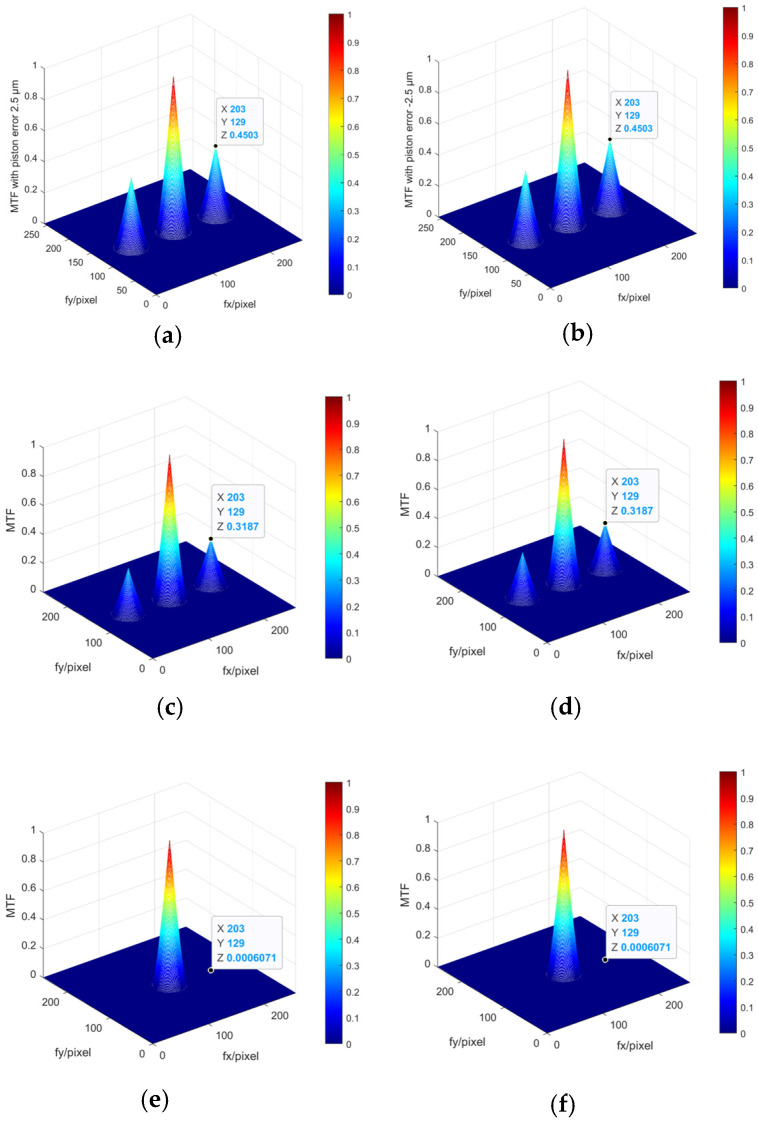
*MTFs* simulated from MATLAB of the two sub-mirrors segmented telescope system (**a**) the system *MTF* with p = 2.5 µm; (**b**) the system *MTF* with p = −2.5 µm; (**c**) the system *MTF* with p = 5 µm; (**d**) the system *MTF* with p = −5 µm; (**e**) the system *MTF* with p = 10 µm; and (**f**) the system *MTF* with p = −10 µm.

Figure 4 shows the values of the *MTF* sidelobes calculated from Equation (10) aimed for the two sub-mirrors segmented system described in Section 2.1. It can be seen that the value of the MTF sidelobe is an even function for the piston errors; the same absolute values of piston errors with opposite signs have the same MTF sidelobes. The MTFs simulated from MATLAB R2021a, as shown in Figure 5 also prove this point. Therefore, this method could not identify the specific spatial relative positions of each sub-mirror and can only measure the absolute value of the piston error.

### 2.3. Using the Resnet Network to Detect the Signs of Piston Errors from Focal Plane Images

Using neural networks to solve the aberrations of optics systems from focal plane image is essentially a process of classifying the degraded images of the focal plane with the aberration coefficient as the label. The Resnet network is essentially a deep learning classification network. It proposes a unique residual module, which realizes identity mapping through short circuit hopping and is more sensitive to data fluctuations, thus it is more suitable for building mapping models from image to data. At the same time, the network solved the gradient loss problem caused by network deepening and effectively improved the classification accuracy. However, when the required detection range is very wide and the detection accuracy is quite high, as for the large aperture and super large aperture segmented telescopes, the number of network classifications increases explosively, which greatly increases the network size and training difficulty. Therefore, the Resnet network in this paper is used to detect the positive and negative signs of piston errors, which can ensure the detection accuracy of piston error signs from the degraded focal plan images with little distinction.

**Figure 6:**
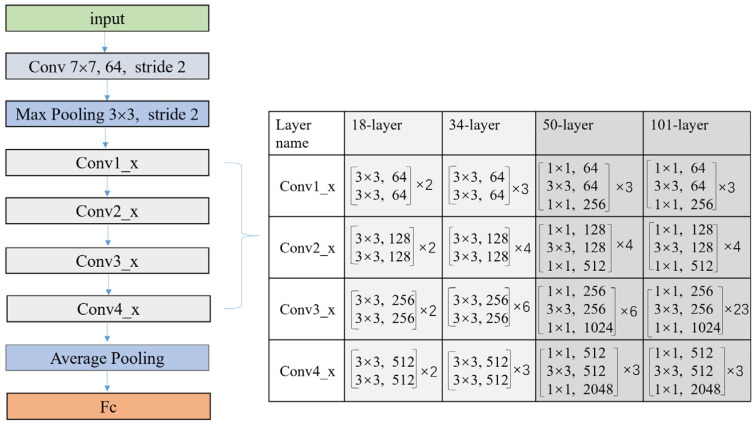
The structural diagram of the Resnet network.

**Figure 7:**
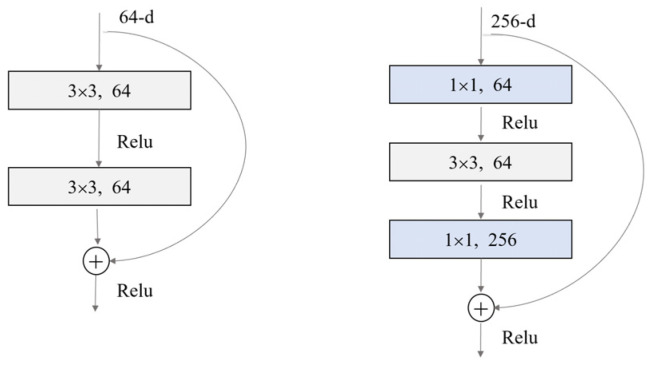
Different residual elements.

The specific Resnet network structure is shown in Figure 6, which includes an input part, an output part, and an intermediate convolution part of four stages. Here, we present four different Resnet networks with depths of 18, 34, 50, and 101; the difference in depth is due to the use of different numbers of residual units in modules from conv1 to conv4. The structure of residual units with different depths is also different. Figure 7 shows two different residual units. Please note that the full connection layer of the netWork needs to be modified to adapt the vector dimension of the output piston error. 

### 2.4. Use BP Network to Detect the Absolute Value of Piston Errors from MTF

According to Equation (10), when the system parameters are determined and the piston errors are given, the sidelobes of the system MTF can be directly obtained. However, when the values of the MTF sidelobes are obtained, it is very difficult to use Equation (10) to solve the piston errors in reverse. Junlun Jiang et al. utilized piecewise quartic polynomials to fit the theoretical relationship, but different polynomial coefficients are needed to be constructed in different piston error detection ranges. Thus, the implementation is complicated, and most importantly their method cannot distinguish the signs of piston error. In our work, a BP network is used to learn the mapping relationship between the absolute value of piston error and the MTF sidelobes. When the network training is completed and the value of the MTF sidelobes is input to the network, the absolute value of the corresponding sub-mirror piston error can be directly output. The reason we use a BP neural network to realize the detection of the absolute value of piston error is that the established theoretical relationship between the MTF and the piston value is quite clear, it is just a mapping relation from data to data, which can be easily modeled by a BP network. 

**Figure 8:**
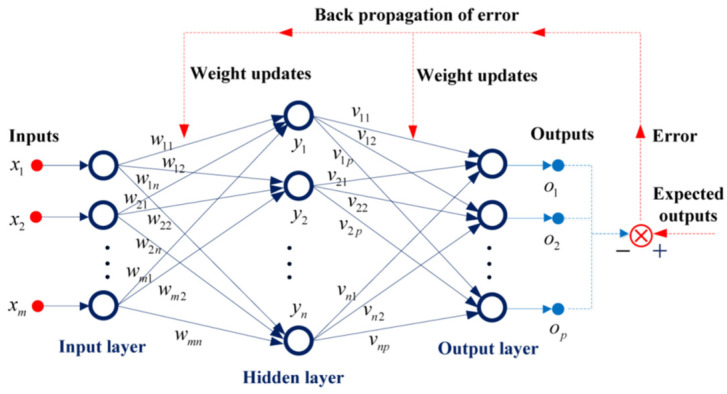
The structural diagram of BP neural network.

The structure diagram of the BP neural network is shown in Figure 8, which contains three layers: an input layer, hidden layer, and output layer. The dark blue circle nodes in each layer represent artificial neurons, which are analogous to axons in a biological brain. The *m*-dimensional vector ***x*** is the input signals, the *n*-dimensional vector ***y*** is the signals of the hidden layer, and the *p*-dimensional vector ***o*** stands for the final output signals. The connection using arrows between neurons illustrates transmitting of the signals from one neuron to next neuron. The receiving neuron can process the signals and then signal to downstream neurons connected to it. The relation between the signals of the receiving neurons and the emitting neurons can be shown as $y_{k}=\varphi \left(\sum_{i=1}^{m}x_{i}w_{ik}-b_{ik}\right)$, where $\varphi \left(\cdot \right)$ is called activation function, *m* is the number of the input neurons, $w_{ik}$ is the weight for the *i*th input signal *x_i_*, and $b_{ik}$ is the bias which used to properly shift the results of this linear transformation. The same relationship also exists between the output signals *o* and the signals *y* in the hidden layer, where $v_{ik}$ represents the weight coefficient. The weights and bias are unknown, and the process of adjusting the weights such that they can learn the appropriate mapping relations between the inputs and the outputs is called learning or training. The training begins with random weights, and the goal is to adjust them so that the error defined by the difference between the expected outputs and the actual network outputs will be minimal.

The BP network algorithm can be divided into two steps: forward propagation and back propagation. In the forward propagation process, the input signal of the input layer is propagated to the output layer through the hidden layer, then the actual outputs are obtained. In the back propagation process, the difference between the actual output and the expected output of the network is taken as the error signal, and the error signal is propagated layer by layer from the output layer to the input layer, and the weights and thresholds of the network are adjusted by a certain algorithm. Through one forward propagation and one back propagation, one update of the network parameters can be realized. The network training process is to continually carry out forward propagation and back propagation and update the network parameters until the error signals become smaller and smaller, finally resulting in the network accurately mapping the relationship between the input and the output.

## 3. Simulation

Using the hybrid network proposed in this paper to detect the piston errors of sub-mirror consists of three steps. Firstly, establish the data sets for network training. Within the coherent length range of the input broadband light, multiple groups of piston errors are randomly generated and loaded onto the corresponding sub-mirrors. By taking the piston errors to the established simulation segmented optical system, the corresponding focal plane degraded images are generated for training the Resnet network, which is used to detect the signs of piston errors. Then, taking the absolute value of the piston errors and the optical system parameters into Equation (10), the values of the MTF sidelobes can be obtained to train the BP network. Secondly, train the hybrid network. The generated focal plane degraded images and the signs of the corresponding piston errors are used as the input and output of the Resnet network, respectively, and with the cross-entropy loss function used as the index function of the network optimization, the Resnet network can be well trained through a gradient-based stochastic optimization algorithm: Adam. Then, the calculated values of the MTF sidelobes and the absolute values of the corresponding piston errors are used as the input and output of the BP network, respectively, and by setting up a specific training algorithm, the BP network can also be well trained. Finally, after the hybrid network training is finished, by taking a degraded focal plane image into the Resnet network, the signs of all sub-mirrors’ piston errors can be obtained. Then, performing the Fourier transform on the focal plane image obtains the values of the MTF sidelobes which can be input to the BP network; the absolute values of all sub-mirrors’ piston errors can be solved. One thing to note here is that the corresponding relationship between the MTF sidelobe and its related sub-mirror should be established in advance, as the absolute values of all the sub-mirrors’ piston errors can be measured simultaneously by one CCD broadband image. Thus, the measurement of the piston error with high precision and a wide detection range can be realized by combining the output of the two networks. 

We first conducted simulation experiment analysis on the segmented telescope system composed of two hexagonal sub-mirrors, as shown in Figure 2. Then, the simulation experiments were processed to multiple sub-mirrors (*n* > 2) segmented telescope system which was composed of four hexagonal sub-mirrors. In the end, we perform some comparation work between our method and the work published by Ma Xiafei et al. in paper [17], since they also used a single wide-band image of a point source to perform piston sensing by a neural network.

### 3.1. Simulation on Two Sub-Mirrors Segmented Telescope System

MATLAB software was used to build the simulation optical system model consisting of two hexagonal sub-mirrors as, shown in Figure 2. The specific system parameters were the same as those set in Section 2.1. During the simulation experiment, the left sub-mirror of the system was set as the reference sub-mirror, and a series of piston errors were introduced on the right sub-mirror. Here, we firstly generated 60,000 sets of piston errors in [−10~10] µm randomly and 60,000 sets of focal plane degradation images were obtained by MATLAB simulation. Then, by taking the corresponding piston errors into Equation (10), we obtained 60,000 sets of the MTF sidelobes values. The generated data sets were divided into three groups, namely the training set, verification set, and test set. The proportion of the three parts was 65%:20%:15%, namely 39,000 groups for training, 12,000 groups for verification, and 9000 groups for testing.

Then, the network training was be processed based on the obtained data sets. Here we built a Pytorch deep learning environment on an Ubuntu server equipped with Intel i7 CPU and Nvidia GeForce 2080 GPU both from the City of Santa Clare, CA, USA, to achieve the training of the hybrid networks. The Resnet network was first trained to predict the signs of piston error. The focal plane degradation images shown in Figure 3 were used as the input of the Resnet network, and the signs of the piston errors were used as the output of the Resnet network, with the label ‘0’ representing positive and the label ‘1’ representing negative. Each network was trained with 300 epochs, the batch size was set as 32, and the cross-entropy loss function was used as the index function. The network parameters were updated by back-propagation and the evaluation of the trained network was realized through the verification set. At the same time, in order to improve the network efficiency, a kind of strategy known as batch normalization was used between convolutional layers to prevent gradient disappearance.

**Figure 9:**
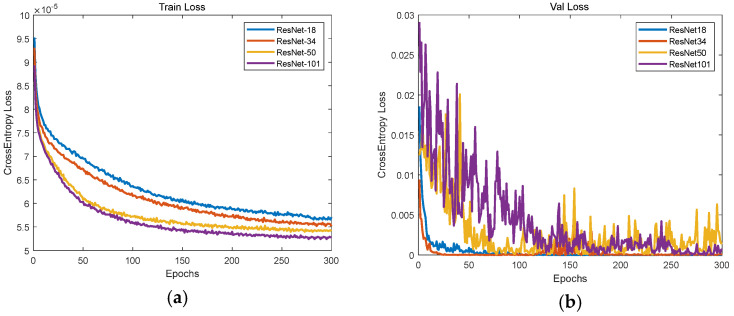
The loss function curves of the four Resnet models: (**a**) Training data set; (**b**) validation data sets.

**Figure 10:**
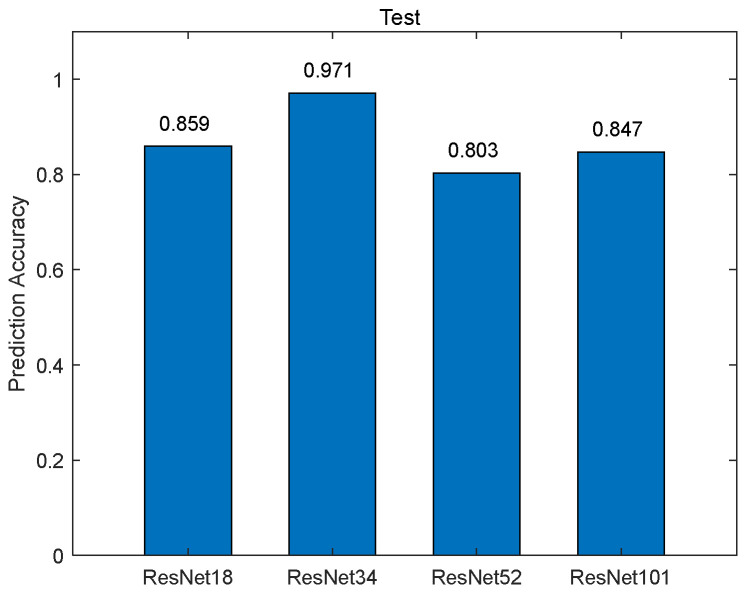
The piston error signs prediction accuracy for the four Resnet models.

Four Resnet networks with different depths, including Resnet18, Resnet34, Resnet50, and Resnet101, were trained here to test their prediction accuracy of the piston error signs. Figure 9 shows the loss functions of the training set and the verification set, where the horizontal axis represents the number of trainings and the vertical axis represents the cross-entropy loss function. We can see that after 300 rounds of training, the loss function gradually declined and finally reached a stable state. From the loss function curve of the training set, the Resnet networks fitting degree gradually increased with the deepening of the network, but from the loss function curve of the verification set, with the deepening of the network, the generalization ability of the networks Resnet50 and 101 were much lower than that of Resnet18 and 34, resulting in the loss function oscillation of the verification data set. Then, we use the test data set to verify the piston error sign prediction accuracy of the four Resnet networks with different depths. The test result is given in Figure 10 where the prediction accuracy of the piston error signs is given by the number of correct predictions in the test set divided by its total number. From Figure 9 and Figure 10, we can see that the Resnet34 network had the highest generalization ability and the highest prediction accuracy. Therefore, the optimal model for the prediction of the signs of piston error for the two sub-mirror segmented system is the Resnet34 network.

**Figure 11:**
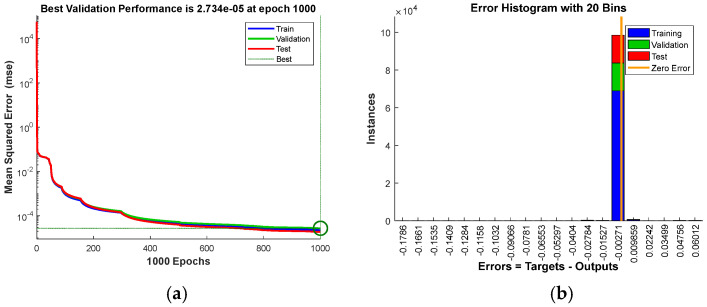
Training results of the BP neural network for the two sub-mirrors segmented system: (**a**) loss function; (**b**) error histogram.

Then, the BP neural network was trained to solve the absolute value of the piston errors. The values of the MTF sidelobes shown in Figure 5 were taken as the input of the network, and the corresponding absolute values of the piston error were taken as the output of the network. The number of neurons in the hidden layer of the BP network was set as 10, the node transfer function of the hidden layer was logsig function, the node transfer function of the output layer was purelin function, and the learning training function was traindx, which is a variable learning rate momentum algorithm. The training results of the network are shown in Figure 11. Figure 11a shows the loss function changing with the number of iterations and Figure 11b provides the error distribution between the expected value and the actual network output value in the form of a histogram. According to the training results, the RMSE between the expected value and the actual output of the network in the training set, the verification set, and the test set are 2.235 × 10^−5^ μm, 2.734 × 10^−5^ μm, and 1.873 × 10^−5^ μm, respectively. It was proved that the BP neural network proposed here can be used to calculate the absolute value of piston error with high precision.

After the hybrid network is well trained, the actual network performance can be tested. Here, another 500 new focal plane images were generated from the simulation optical system for testing. In order to approximate the actual imaging environment, Gaussian distribution noise with a mean of 0 and a variance of 0.05 was introduced into the simulated *PSF* images. Furthermore, since tip–tilt errors cannot be completely corrected, the tip–tilt errors were also added to each sub-mirror during the generation of the focal plane degradation image, where the total RMSE value of the added tip–tilt errors was 0.01 λ. Since we only considered the co-phase errors like Keck where all sub-mirrors were assumed to have the same shape and be perfect without high-order aberrations except pistons and tip–tilts, higher order aberrations of each sub-mirror were not considered here. 

**Figure 12:**
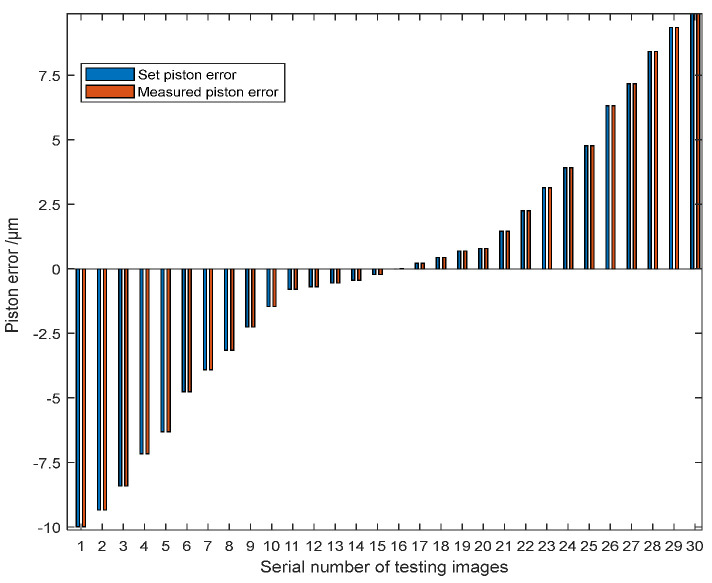
Piston error measurement results by the proposed hybrid network.

Figure 12 shows 30 groups of the experiment results randomly selected from the whole 500 groups. The piston error detection accuracy was given by the difference between the measured piston error and the set piston error. According to the error analysis, the RMSE of the difference values of the 30 experimental results is 1.26 nm where the RMSE is defined as $RMSE=\sqrt{\frac{1}{N}\sum_{n=1}^{N}Difference_{n}^{2}}$; *N* is the number of experiment groups. 

**Figure 13:**
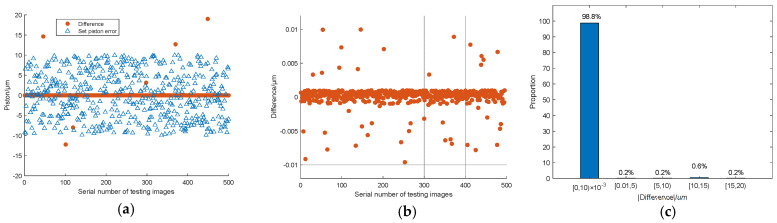
Piston error detection results of the 500 test sets for the two sub-mirror segmented system: (**a**) the whole detection results of the 500 test sets; (**b**) detection results after removing the 6 sets of outliers; (**c**) statistical histogram distribution of the piston error measurement difference.

Figure 13 shows the piston error detection results of all the 500 groups of simulation experiments, among which the difference in the detection results within 494 groups is 10 nm, and the RMSE of the 494 groups is 1.76 nm. The left 6 groups of difference values of the piston error detection results are very large, far greater than the mean difference values of the 494 groups of piston error detection results. This is because the signs of piston errors were predicted wrongly by the Resnet network, and the measurement error was basically twice of the set piston error. Figure 13a shows the piston error measurement results of all the 500 groups of simulation experiments, Figure 13b shows the measurement errors of these 494 groups in the form of a scatter plot, and Figure 13c takes the absolute value of measurement error as the horizontal coordinate to give the statistical results of the 500 groups of piston error detection experiments. It can be seen that the detection range of this method is very wide, ranging from −10 μm to 10 μm, reaching the whole coherence length of the input broadband light. The measurement accuracy is very high, and the probability of measurement error less than or equal to 10 nm is as high as 98.8%. Under the characteristics of our workstation, the time consumed by the Resnet 34 network to detect the signs of piston error from a degraded focal plane image was 3.1 ms, the time consumed of the BP network to detect the absolute value of one piston error from the MTF sidelobe obtained by Fourier transformation of the degraded focal plane image was 0.45 ms. So, the detection time of the proposed algorithm is quite fast as long as the hybrid network is well-trained. 

### 3.2. Simulation on Four Sub-Mirrors Segmented Telescope System

In this part, we use a four sub-mirror segmented telescope system as an example to test the performance of the proposed algorithm on detecting the piston errors of a multiple sub-mirrors (*n* > 2) segmented telescope system at one time. When using the Resnet network to detect the signs of piston errors of the multi-submirrors, the output of the Resnet network was set to 2*^(N−1)^*, which corresponds to the piston error signs of all sub-mirrors (except the reference sub-mirror). When the BP network was used to solve the absolute value of the piston errors of multiple sub-mirrors through the MTF sidelobes, according to paper [16], we used a segmented telescope system composed of *N* sub-mirrors, there are *N^2^* sub-MTFs. In the spatial frequency domain, the *N* sub-MTFs overlapped at the position where the center spatial frequency was zero to form the central peak, while the other *N(N − 1)* sub-MTFs distributed around the central peak to form the sidelobes. Every pair of sub-mirrors produced a pair of MTF sidelobes; the sidelobes symmetrically distributed on both sides of the central peak. If all of the sidelobes did not overlap, their amplitudes could be obtained at the same time by one CCD image, hence the absolute value of the piston errors of all sub-mirrors can be retrieved at the same time by inputting the peak height of the sub-MTFs corresponding to each sub-mirror into the trained BP network. Combining the outputs of the two networks, the piston errors of all the segmented sub-mirrors can be solved at one time using a focal plane degradation image.

**Figure 14:**
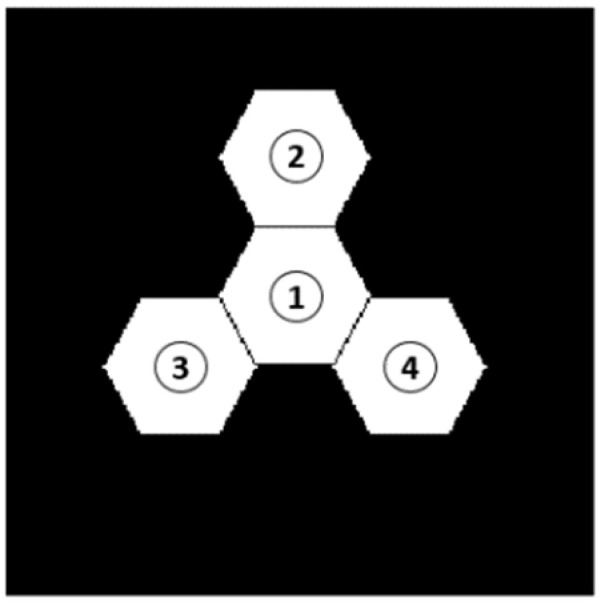
Segmented telescope model composed of four sub-mirrors.

**Figure 15:**
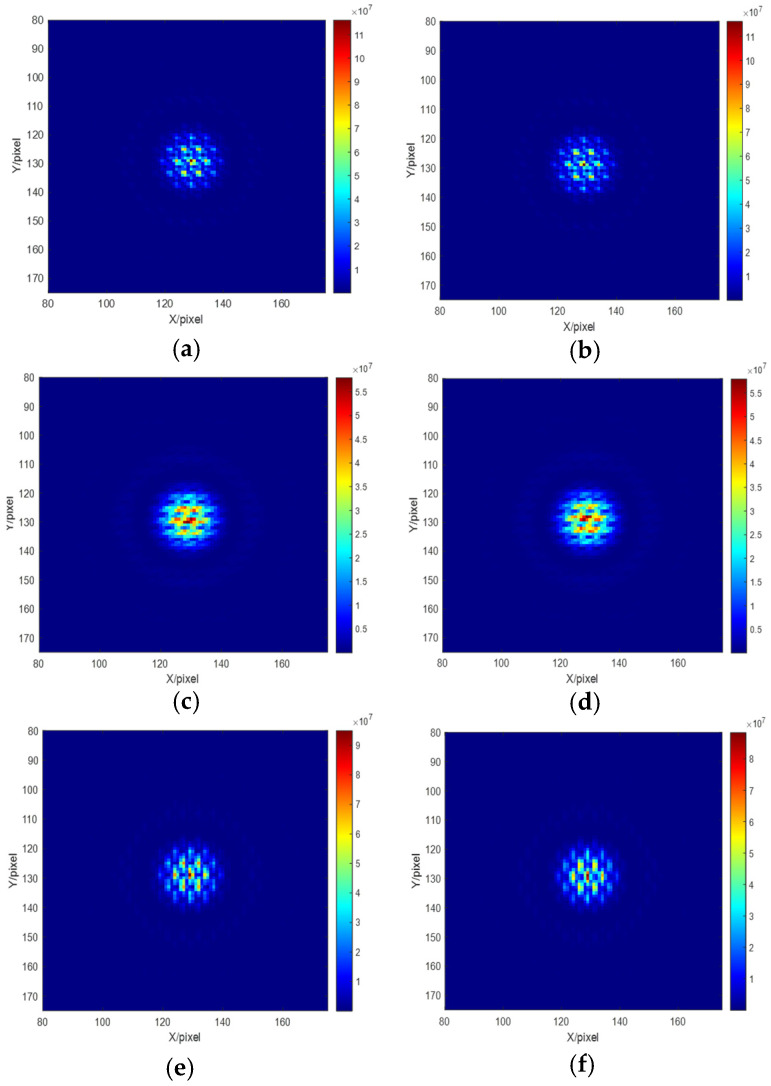
Degraded focal plane images of the four sub-mirror segmented system: (**a**) [p2 p3 p4] = [−2.5 0 5] μm; (**b**) [p2 p3 p4] = [2.5 0 −5] μm; (**c**) [p2 p3 p4] = [4 −7.5 10] μm; (**d**) [p2 p3 p4] = [−4 7.5 −10] μm; (**e**) [p2 p3 p4] = [9 0.6 2.75] μm; (**f**) [p2 p3 p4] = [−9 −0.6 2.75] μm.

The established simulation model of a four sub-mirrors segmented telescope system in MATLAB is shown in Figure 14. The reason why the system model was built like this was to prevent the MTF sidelobes from overlapping. Here, the wavelength of the input broadband light was also centered at 632.8 nm with a 20 nm bandwidth. The No. 1 sub-mirror was set as reference pupil and the piston errors randomly generated between −10 µm and 10 µm were introduced on sub-mirrors No. 2, 3, and 4, thus the degraded focal plane images with different piston errors could be obtained. Figure 15 shows several degraded images on the focal plane corresponding to the several sets of introduced piston errors on multiple sub-mirrors. 

During the simulation experiment, 60,000 sets of piston errors were randomly generated and introduced into the simulation optical system; 60,000 groups of focal plane degradation images can be obtained. The focal plane degradation image was used as the input of the Resnet network, and the signs of the piston errors of the 3 sub-mirrors were used as the output. Here, the Resnet network output was divided into eight categories. When constructing the labels of network outputs, we used 0 to indicate a positive sign and 1 to indicate a negative sign, which is similar to the binary encoding process and is shown in the Table 1. The other setting parameters and training process of the Resnet network were the same as those of the two sub-mirror system, which will not be repeated here. 

**Figure 16:**
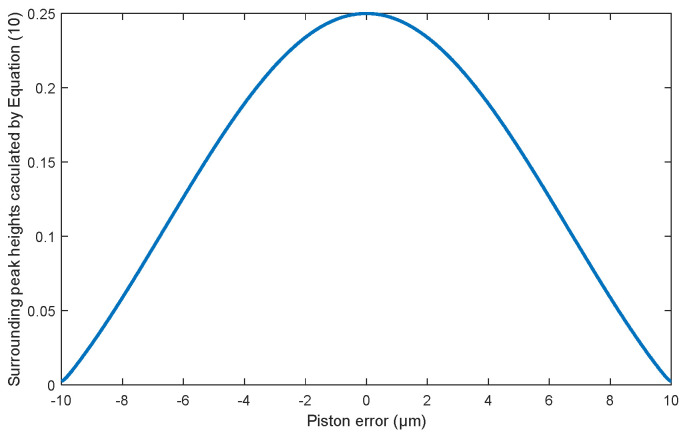
The *MTF* sidelobes with different piston errors for the four sub-mirror segmented system.

When training the BP network, we took the number of sub-mirrors, *n* = 4, the wavelength of the input broadband light, and the 60,000 sets of piston errors generated randomly into Equation (10). We used the modulus values of the *MTF* sidelobes calculated directly from Equation (10) as the input matrix, while the corresponding piston errors were used as the output matrix of the training network. Figure 16 shows the relationship between the modulus values of the *MTF* sidelobe directly calculated by Equation (10) and the piston error of sub-mirror in the form of a curve. The obtained 60,000 data sets were used to train the BP network. The parameter setting and the training process of the BP network were the same as that of the two sub-mirrors segmented optical system, which also will not be repeated here.

**Figure 17:**
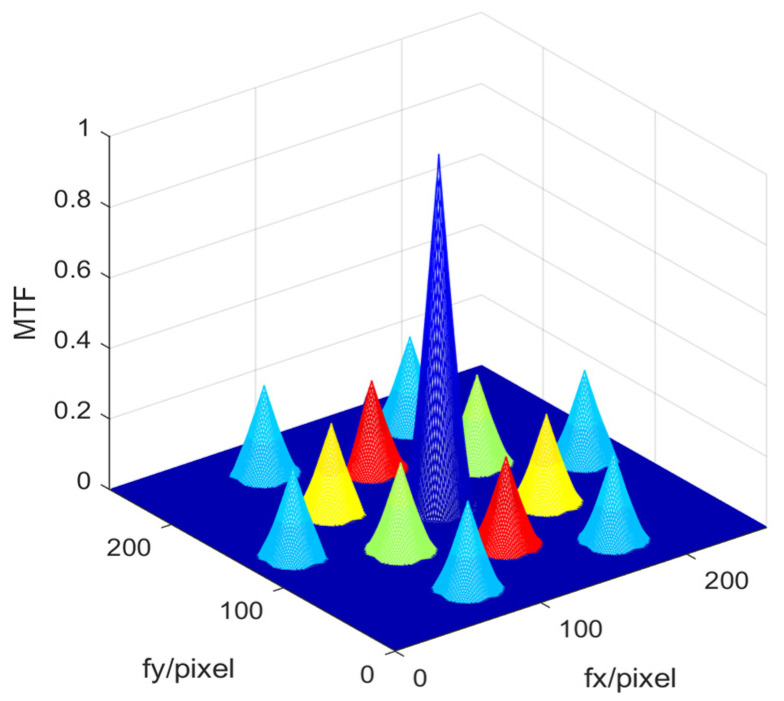
The *MTF* of the four sub-mirror segmented system with color-marks.

In order to solve the absolute value of the piston errors of the multiple sub-mirrors simultaneously from one focal plane image based on the well-trained BP network, the mapping relationship between sub-*MTFs* and their corresponding sub-mirrors had to be established in advance. The system *MTF* of the four sub-mirrors segmented optical system in this experiment included a central main peak and 12 (N (N − 1) = 4 × (4 − 1) = 12) sidelobes, which is shown as Figure 17 with color-marks. When sub-mirror No. 1 was set as the reference mirror, sub-mirror No. 2 produced the red sub-*MTF*s, sub-mirror No. 3 produced the green sub-*MTFs*, and sub-mirror No. 4 produced the yellow sub-*MTFs*. The six peripheral blue sub-*MTFs* were modulated by the piston errors of the other two sub-mirrors simultaneously, except the reference mirror, which cannot be used to measure the piston error of each sub-mirror. In order to ensure the effectiveness of the proposed method, one of the most important things was to avoid any overlap of the *MTF* sidelobes, which were formed by each pair of sub-waves sampled by the corresponding pair of the sub-apertures during the establishment of the mapping relationship. Thus, the arrangement of multiple apertures should be designed scientifically. The detailed arrangement rule can refer to paper [16]. It should be noted that the relationship between the *MTF* sidelobes and the absolute value of the piston error of each sub-mirrors was the same (as shown in Figure 16), thus it was not necessary to conduct training for all three sub-mirrors at one time, but to use one data set to train a single BP network. Then, by inputting the modulus of sub-*MTF* corresponding to each sub-mirror directly, the piston error absolute value of each sub-mirror could be obtained. This can reduce the difficulty of network training and improve the detection accuracy.

After the hybrid network is well trained, the actual performance of the network should be tested. We randomly generated multiple sets of piston errors in the range of [–10~10] um and introduced them into sub-mirrors No. 2, 3, and 4 separately, then the focal plane degradation image could be obtained. In order to be closer to the real imaging situation, Gaussian noise with mean 0, variance 0.05, and tip–tilt errors with RMSE 0.01 λ were added to the generated the focal plane degradation images.

**Figure 18:**
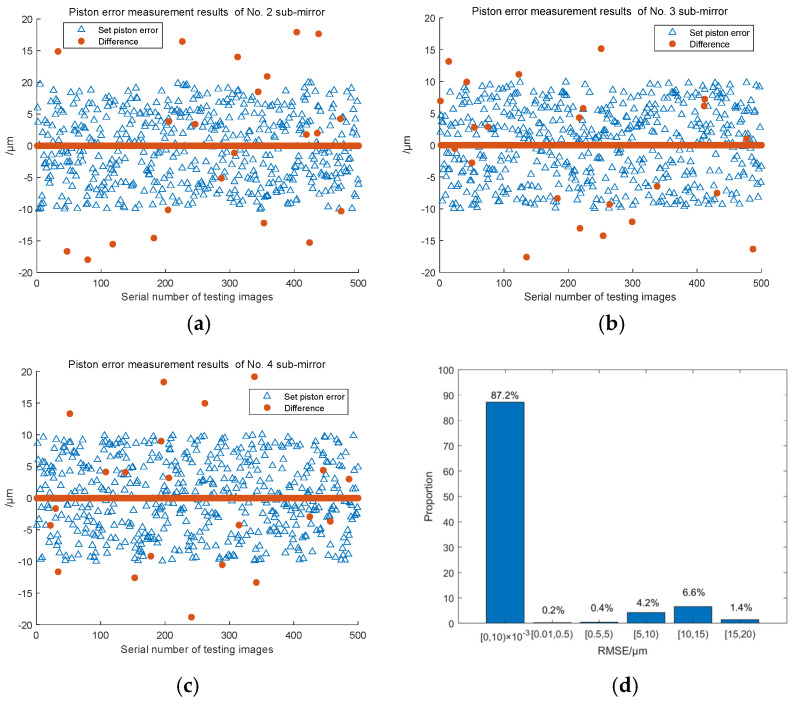
3 Piston error detection results of the 500 test sets for the four sub-mirror segmented system. (**a**~**c**) Piston error detection results of sub-mirror No. 2, 3, and 4, respectively; (**d**) the RMSE values histogram distribution of the 3 sub-mirrors’ piston error detection results.

We also generated 500 focal plane images of the system for testing, and the test results are shown in Figure 18. The piston error detection results of sub-mirror No. 2, 3, 4, are shown in Figure 18a–c, respectively, Figure 18d shows the distribution histogram of the RMSE values of all three sub-mirrors’ piston errors detection results in the 500 groups. It can be seen that with the increasement in the number of sub-mirrors, the classification number of the Resnet network became greater, and the detection accuracy of the signs of the piston errors decreased, but the detection accuracy of the BP network had no change. The probability of a measurement error less than or equal to 10 nm could still be maintained above 85%.

### 3.3. Comparation Work

Finally, we compared our method with the work published by Ma Xiafei et al. in paper [17], because they also used a single wide-band image of a point source to perform piston sensing by neural network, where a DCNN network was used to directly learn the mapping relationship between the focal plane degradation images and the piston errors of sub-mirrors. The typical architecture of DCNN comprises one input layer, several convolutional layers, pooling layers, fully connected layers, and one output layer, among which convolutional layers together with pooling layers play a role as feature extractors. The DCNN Ma et al. used has 26 layers, the detailed structure of which can be referred to in Figure 2 of paper [17]. The main difference between Ma’s method and ours is that they did not establish the precise theoretical relationship between *MTF* and piston error based on Fourier optics, thus the piston error detection was realized based on a single neural network. This increased the training difficulty of the network and could not guarantee the high piston error detection accuracy. 

**Figure 19:**
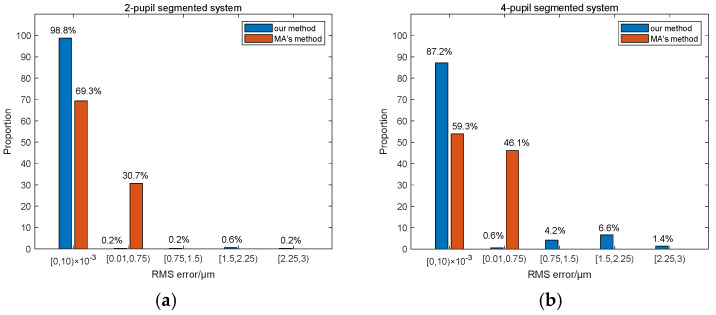
Comparative experimental results of piston error detection accuracy between Ma’s and our methods: (**a**) for the two sub-mirrors segmented system; (**b**) for the four sub-mirrors segmented system.

The comparison experimental results aimed for the two-pupil segmented systems and the four-pupil segmented systems are shown in Figure 19. It should be noted that the relevant experimental parameters must be set to the same during the comparison. Here, we used the same parameters as Ma’s work for both the two-pupil segmented system and the four-pupil segmented system, including the 500–600 nm broadband input light, 500 mm focal length, and 1.67 μm pixel size of the CCD. It can be seen that the piston error detection accuracy of the method proposed by us is generally higher than that of the method proposed by Ma et al. This is because we constructed the theoretical relationship between the system *MTF* and the piston errors and used the modulus of the *MTF* sidelobes as the network input, while Ma et al. directly used the focal plane image as the input of the network. However, the maximum value of piston error detection difference of our proposed method is much larger than that of Ma’s method. This is because when using the Resnet network to detect the signs of the piston error wrongly, the detection difference value was almost twice that of the set piston error value. So, how to improve the detection accuracy of the signs of piston error is quite important in our next work.

## 4. Discussion

This paper proposed a piston error detection method based on a hybrid neural network, it can realize a wide-range and high-precision measurement of all sub-mirrors’ piston errors by using a single wide-band image of a point source observation target at one time. Its detection range can reach the entire coherent length of the input broadband light. For the 2-pupil segmented system, the proportion of detection accuracy within 10 nm is 98.8%, and for the 4-pupil segmented system, the proportion of detection accuracy within 10 nm is 87.2%. Due to the establishment of the theoretical relationship between the piston errors of the sub-mirror and the *MTF* of the system, each network’s structure in the hybrid network is relatively simple, which greatly reduces the network size and training difficulty, and ensures that the proposed method is simple to implement. By using the method proposed in this paper, the piston error detection of the segmented optics system no longer needs to be divided into coarse phasing and fine phasing where different hardware devices are needed. This greatly reduces the detection complexity and cost and provides a relatively simple and feasible piston error detection method for the large aperture segmented telescope optics system.

## Figures and Tables

**Table 1:** Resnet network output label.

Input	Positive and Negative Signs of Each Sub-Mirror	Classification Code	Label
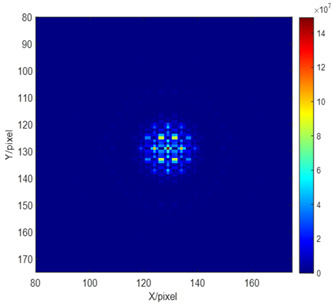	+ + +	000	0
	+ + −	001	1
	+ − +	010	2
	+ − −	011	3
	− + +	100	4
	− + −	101	5
	− − +	110	6
	− − −	111	7

## Data Availability

Data is unavailable due to privacy.
